# February precipitation in the wintering grounds of the lesser whitethroat, *Sylvia curruca*: is it a cue for migration onset?

**DOI:** 10.1098/rsos.160755

**Published:** 2017-02-15

**Authors:** Irith Aloni, Shai Markman, Yaron Ziv

**Affiliations:** 1 Spatial Ecology Laboratory, Department of Life Sciences, Ben-Gurion University of the Negev, Beer Sheva, Israel; 2 Department of Biology and The Environment, Faculty of Natural Sciences, University of Haifa - Oranim, Oranim, Israel

**Keywords:** spring migration, climate change, wintering grounds, long-distance migration, *Sylvia curruca*, lesser whitethroat

## Abstract

Numerous studies report shifts in bird migration phenology, presumably owing to global warming. However, most studies focus on migration patterns in the Northern Hemisphere. In this study, we investigated associations between weather conditions in African wintering grounds of the lesser whitethroat, *Sylvia curruca,* and spring arrival time in Eilat, Israel. Using multivariate regression models, we analysed a 30-year dataset in order to examine correlations between median springtime arrival and 46 climate variables of the wintering quarters. The model obtained exhibited a highly statistical fit, involving mean precipitation in February and March with negative effects and number of wet days during November–February. February precipitation levels were also the major factor associated with the interquartile range of arrival time. Interestingly and contrary to published results, annual or seasonal precipitation showed no correlation with spring arrival time, nor did temperature. Moreover, winter in this region falls into dry season with negligible rainfall quantities. Hence, it is unlikely that precipitation effect on habitat productivity is a driving force of migration, as suggested by other studies. Instead, we propose that precipitation in February acts as a cue for the birds, indicating the approach of spring and migration time.

## 1. Introduction

Changes in bird migration phenology are well established and commonly linked to global warming. Many migratory bird species have been reported to advance spring arrival at breeding grounds, most likely because of rising temperatures [1–5]. Other studies revealed delayed arrival, possibly because of winter droughts, which caused shortage of necessary food resources for pre-migratory fuel deposition [3,5–7].

Exact timing of spring arrival at breeding grounds is crucial; birds must arrive early enough to occupy a territory, find a mate and match egg production and hatching with peak food abundance. Yet, too early an arrival may risk survival owing to possible freezing temperatures and lack of food [1,5,8–9]. Timely arrival is particularly tricky for long-distance migrants in light of the changing climate, as changes are not uniform across different geographical regions [10].

Onset of migration has been postulated to be under rigid endogenous control and synchronized by photoperiod and internal clocks [9,11]. However, other studies suggest phenotypic plasticity, as inflicted by shifts in arrival time at European staging and breeding grounds of various species [4–5].

Jonzén *et al.* [3] reported delayed arrivals of several long-distance migrants in Capri, Italy, on springs with high values of North Atlantic Oscillation Index (NAOI; an index that describes fluctuations in atmospheric pressure over the North Atlantic Ocean). High NAOI values have a negative effect on precipitation in northeastern and southwestern Africa, which may imply food shortage for pre-migratory fuel deposition. Both *et al*. [10] found that arrival time and breeding date of the pied flycatcher, *Ficedula hypoleuca,* depend on temperatures in both North African staging grounds and in The Netherlands breeding grounds. Halkka *et al*. [12] revealed negative correlations between arrival time to Finland and temperatures in southern and central Europe along the presumed migration route. Marra *et al*. [6] showed that the interval in median capture dates between the Gulf Coast of Louisiana and two ringing stations, in Pennsylvania and Ontario, was inversely correlated with temperature for 15 species.

These studies, along with several others, indicate that environmental factors such as temperature and precipitation are related to phenological shifts in migration. However, mechanisms controlling these shifts are controversial. Some researchers suggest that environmental factors modify migration rate once triggered while others claim that environmental factors trigger migration onset directly [4–5,9,13–15].

Various studies support the different views. Saino *et al*. [16] revealed that spring arrival dates of nine trans-Saharan migratory species to Capri, Italy, were negatively correlated with temperatures in the Sahel wintering grounds and rainfall in North African passage areas. They concluded that migration phenology is mostly a result of phenotypic adjustment to meteorological conditions in Africa during the wintering period. Studds & Marra [15] showed that annual variation in tropical rainfall and food resources are associated with marked changes in the timing of spring departure of the same individuals of the American redstart, *Setophaga ruticilla*. They stated that this finding challenges the idea that photoperiod alone regulates the onset of migration, providing evidence that intensifying drought in the tropical winter could hinder adaptive responses to climatic warming in the temperate zone.

One major limitation to the understanding of the mechanisms operating in the modification of migration timing is the lack of sufficient research conducted at wintering grounds of long-distance migrants. Most studies of Palaearctic migrants examined bird arrival at European breeding or staging grounds. Only a few investigated the effect of climate at African wintering grounds on timing of spring migration, and fewer studied these effects *en route*, before arrival to Europe [5,13,17–18]. Since birds may spend considerable time at stopover sites, arrival time at European grounds may not well inflict timing of departure from wintering grounds [19–21]. Thus, information regarding timing of arrival at stopover sites further south is absolutely essential.

In this study, we address the question of the effect of climate conditions in African wintering grounds on birds' arrival time to Eilat, Israel. Based on earlier propositions, we test three previously mentioned hypotheses. Our first hypothesis posits that high temperatures at wintering grounds induce early departure as they may indicate to the birds an advanced spring [1]. Our second hypothesis maintains that harsh weather conditions (i.e. extreme temperatures and/or low precipitation levels) at the wintering grounds induce early departure owing to habitat unsustainability [22]. Finally, in contrast to the second hypothesis, our third hypothesis states that harsh weather conditions at the wintering grounds prevent timely departure because of difficulty to acquire sufficient fat reserves necessary for migration [7,23].

We used the lesser whitethroat, *Sylvia curruca*, as a case study, as it is a common passerine on which a large dataset is available. Most European populations of the lesser whitethroat migrate back north through the Eastern Mediterranean Flyway which narrows down to a ‘bottleneck’ path when approaching the Middle East region [24,25]. As a result, numerous birds pass through this very narrow region during migration. Eilat, located at the very southern tip of Israel immediately after crossing of the Sahara desert barrier, is a major stopover site. Arrival time to Eilat, located relatively close to the wintering grounds, is a much better reflection of departure time from wintering grounds than arrival time in Europe, 1500–4500 km further north. Hence, analysis of possible relationships between climate conditions at African wintering grounds and springtime arrival in Eilat may offer some new insights as to questions regarding possible effects of climate change on spring migration of the lesser whitethroat.

## 2. Material and methods

### 2.1. Study species

The lesser whitethroat, *S. curruca*, is unique among Sylvidae as the major balk of its European populations migrates north through the eastern part of the Mediterranean [24,25]. First migrants may arrive in Eilat, Israel, in mid-February and arrivals continue until May or early June. Daily peaks exceed 2000 individuals in Eilat alone, and in the Arava Valley, a stretch of 50 km to the north of Eilat, estimates on peak days stand at 10 000 birds [26]. Bird data for the years 1984–2013 were obtained from the International Birding and Research Centre in Eilat (IBRCE) and included 26 841 ringing observations of *S. curruca*.

### 2.2. Data collection

Climate data of wintering grounds was downloaded from the University of East Anglia, UK, Climate Research Unit website [27]. For the wintering and breeding grounds, we followed the species distribution map available at Birdlife International website [28]. We applied this map to the climate dataset to extract the region of interest. The final winter climate set included 1422 grid points with monthly measurements of environmental variables containing components of air temperature, precipitation, humidity, number of wet days and cloudiness. This was used to create a set of 46 climatic variables, which represented various statistical location and spread components of the grid points of each variable (e.g. mean, median, percentiles, proportion of sites with a certain trait, etc. see the electronic supplementary material, appendix SI).

### 2.3. Data analysis

For explanatory variables, we used climate conditions during the latter part of the wintering season (December to March) as well as annual and seasonal precipitation variables. The latter may have a long-lasting effect through primary production and availability of water bodies which facilitate insect proliferation [7,9,17,29,30]. To assess arrival time, we defined 1st February as day number 1 of spring arrival, and used this date as a baseline reference (in a variable referred to, from here on, as *Calenday*). Median arrival time (*Median Calenday*) and interquartile range (*IQR*—the range of time during which the central 50% of birds arrived) were calculated for each year.

Correlations between the 46 original climate variables with *Median Calenday* and with *IQR* were examined (electronic supplementary material, appendix SI). Of these, variables that were highly correlated with the response variables (|*r*| > 0.35) were used for regression analyses. We applied stepwise regressions of various kinds, using this subset of climatic variables, to analyse *Median Calenday* and *IQR*. The best fitting models were derived. Criteria used for model selection included a combination of Akaike Information criteria (AIC) and Mallow's C_p_ criteria.

R software was used for all statistical analyses [31]. The analyses programs were written from scratch (using available commands within the R environment).

## 3. Results

**Figure 1.**
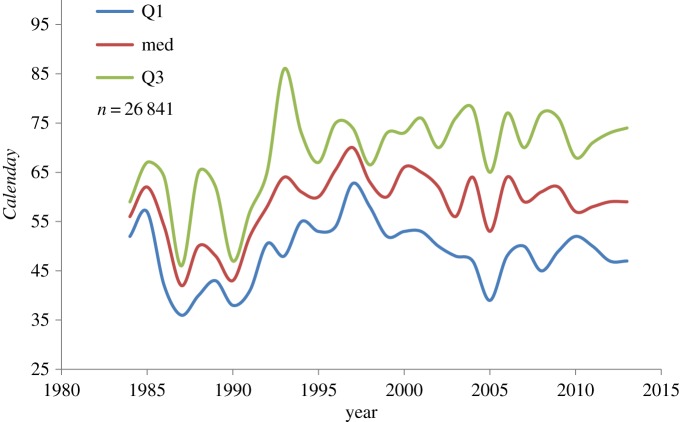
Quartiles and median spring arrival day (*Calenday*) of the lesser whitethroat, *Sylvia curruca,* to Eilat during 1984–2013.

Patterns of median spring arrival (*Median Calenday*) along time exhibited strong fluctuations during the 1980s and early 1990s with increasingly delayed arrival time. In the second half of the studied period (since 2000), the trend shifted to a gradually advanced arrival time and considerably smaller fluctuations among years (figure 1). The interquartile arrival time was rather narrow during most of the first half of the study period, and then expanded considerably (from a 7 day span in 1984 to 27 days in 2013; figure 1).

The best fitting models for *Median Calenday* and *IQR* were surprisingly simple and highly statistically significant, involving three and two climate variables, respectively (tables 1 and 2). Both models fitted well with adjusted *R*^2^-values of 0.64 and 0.58, respectively. In both models, precipitation in February was the most influential explanatory variable. The *Median Calenday* model also involved mean precipitation in March and average number of wet days during November–February. The coefficient estimates for precipitation mean in February and March were negative (table 1). No temperature related variable nor any seasonal or annual precipitation variables (except for proportion of sites with yearly accumulated precipitation above 200 mm) were correlated with *Median Calenday* (electronic supplementary material, appendix SI).

**Table 1.** Best fitting model for median of *Calenday*. (Mar.Mean.Pre, mean precipitation in March over 1422 grid sites of wintering grounds; Feb.Mean.Pre, mean precipitation in February over 1422 grid sites of wintering grounds; Mean.Wet.NF, mean number of wet days during November to February over 1422 grid sites of wintering grounds. ***p < 0.001.)

median *Calenday* ∼ Mar.mean.Pre + Feb.Mean.Pre + Mean.Wet.NF				
coefficients:	estimate	s.e.	*t*-value	Pr(>|*t*|)
(intercept)	50.9276	4.3539	11.697	7.37 × 10^−12^***
Mar.Mean.Pre	−0.4466	0.1644	−2.716	0.0116*
Feb.Mean.Pre	−1.6345	0.2857	−5.720	5.08 × 10^−6^***
Mean.Wet.NF	34.4487	7.1930	4.789	5.86 × 10^−5^***
multiple *R*^2^ = 0.6758	adjusted *R*^2^ = 0.6384			
*F*_3,26_ = 18.06	*p*-value = 1.53 × 10^−6^***

**Table 2.** Best fitting model for IQR of *Calenday*. (Feb.Median.Pre, median precipitation in February over 1422 grid sites of wintering grounds; Feb.Median.Pre^2, squared median precipitation in February; Acc.Median.Pre.MF, median of accumulated annual (March to February) precipitation over 1422 grid sites of wintering grounds. *p < 0.05; **p < 0.01; ***p < 0.001.)

IQR ∼ Feb.Median.Pre + Feb.Median.Pre^2 + Acc.Median.Pre.MF				
coefficients:	estimate	s.e.	*t*-value	Pr(>|*t*|)
(intercept)	−0.008507	5.159415	−0.002	0.99870
Feb.Median.Pre	−38.524487	16.322482	−2.360	0.02605*
Feb.Median.Pre^2	44.795464	13.553401	3.305	0.00277**
Acc.Median.Pre.MF	0.083882	0.024366	3.443	0.00196**
multiple *R*^2^ = 0.6217	adjusted *R*^2^ = 0.5781			
*F*_3,26_ = 14.24	*p*-value = 1.094 × 10^−5^***			

As for the *IQR* model, February median precipitation was by large the major variable involved. The relationship was quadratic, and the coefficient estimates of the first- and second-order components had opposite signs, the first being negative and the second positive (table 2). A closer examination of the model function behaviour revealed that at high values of February precipitation *IQR* increased, whereas at lower values *IQR* did not change much (figure 3). The other factor involved in this model was annual (March to February) precipitation, with a positive coefficient estimate, indicating an increase in *IQR* with increased annual precipitation (table 2).

## 4. Discussion

In this research, we studied possible effects of climate conditions at African wintering grounds of *S. curruca* on their spring arrival time to Eilat, Israel. All of our hypotheses relied on previous propositions found in related migration studies. A surprisingly high correlation was discovered between February and March precipitation and the species spring arrival time. We found that higher February (and March) precipitation levels indicate early migration as well as larger variability among individuals in timing of migration. Additionally, we found that the pattern of spring arrival time fluctuates along time with a switch of directions from a progressively delayed arrival during the first half of the study period, to a gradually advanced arrival on the second half. This latter observation suggests that shifts in migration phenology are not unidirectional. Numerous studies tie advanced spring migration with globally rising temperatures [1,3,5,14,18]. However, our result does not provide support for this proposition, not only because of lack of any correlation between arrival time and temperature at the wintering grounds, but also because of the direction switch in arrival time, while the trend of global temperature rise has remained constant in recent decades [5,8]. This result, hence, leads to the rejection of our first hypothesis, which predicted advanced springtime arrival in response to increasing temperatures at the wintering grounds.

Many authors have suggested that shifts in spring arrival time are a consequence of recent micro-evolutionary adaptations to climate change [1,3,29,32]. However, the observed switch and considerable fluctuations along time do not support this notion. Rather, our results seem to support the assessment that phenotypic plasticity drives seasonal fluctuations, possibly through external fine-tuning of internal clocks.

The model for median spring arrival time in Eilat strongly suggests that precipitation levels in February and, to a lesser extent, in March, are major factors shaping timing of migration of the lesser whitethroat. In spite of the lack of knowledge as to the precise location of wintering sites and, hence, the very large area included in analysis (approx. 2 × 10^6^ km^2^), the model fit is very high (table 1). Pearson correlation between median arrival day and February mean precipitation is −0.54, much higher than that of any other single variable analysed (electronic supplementary material, appendix SI). The negative character of the correlation and, subsequently, the negative coefficient estimates in the model, indicate that higher February and March precipitation levels are associated with earlier departure.

**Figure 2.**
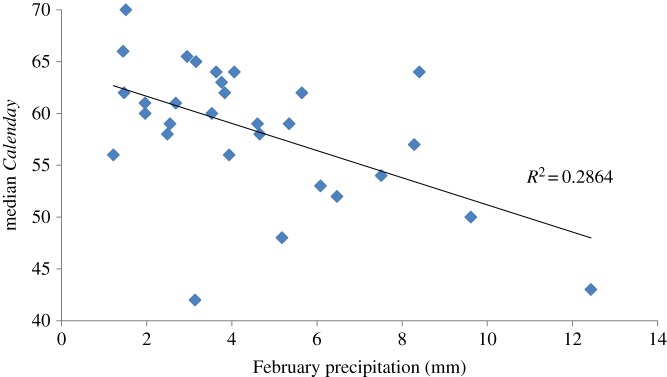
Median arrival day (*Calenday*) of the lesser whitethroat, *Sylvia curruca,* to Eilat as a function of February mean precipitation in Africa, 1984–2013.

Studies relating shifts in spring migration phenology to precipitation levels at wintering grounds usually denote the effect of precipitation on food availability. High precipitation levels may increase resource abundance prior to departure, which may accelerate fat deposition rate and hence facilitate earlier migration [4,22,29,33–37]. However, the period of October to April is a dry season in the lesser whitethroat's wintering grounds with hardly any rainfall at all [9,38]. Throughout the 30 years of study, average February precipitation levels in this region ranged between a mere 1.2 and 12.4 mm (median 3.8 mm; figure 2). Additionally, the rainy season in this area is still a few months away, so it seems unlikely that a few drops of rain would trigger any potential food resource proliferation [4].

Aside from February and March precipitation, only 5 out of 23 variables representing precipitation and humidity were correlated with median arrival time. In particular, no correlation was found with either annual or seasonal rainfall (electronic supplementary material, appendix SI). Of the five variables mentioned, *Number of Wet Days in February* demonstrated the very same trend expressed in the *Median Calenday* model by February precipitation. The other four variables included *Number of Wet Days in November–February* (included in the model), *Mean Number of Wet Days in January* (which is part of the previous variable), *Proportion of Sites with above 200 mm of annual rainfall*, and *Mean Precipitation in January*. These variables most likely reflect some threshold for basic survival. This supposition is supported by the positive correlation sign of these four variables, which suggests delayed migration with increased variable values. That is, if these variables values are low, survival would be low, and the supposedly stronger survivors would take off early, while the weaker would not survive for plausible later migration. Additionally, this result precludes the possibility of positive effect of rain on early fat deposition capabilities through effect on food abundance (otherwise, negative correlations would have been expected). All of these results imply that our second and third hypotheses, which relate to the effect of harsh weather conditions at the wintering grounds on timing, fail to explain the observed results.

Our findings may, hence, imply a completely different process than those suggested for this system so far. A rain event towards the end of dry season may indicate to the birds the onset of season change. This possibility is supported by a meteorological shift and reverse of directions of the two major African air streams in January. This meteorological switch shifts the movement of rains back north, which would bring first drops to the lesser whitethroat's wintering grounds around February or March [9,38]. Hence, it is reasonable to deduce that February precipitation serves as an environmental cue of globally changing seasons. As these meteorological patterns are most likely a prehistoric phenomenon, an evolutionary adaptation may have evolved in order to fine-tune internal migration onset to fluctuations in timing of seasonal weather change.

It should be noted that rain events in arid and semi-arid regions, such as the wintering grounds of the lesser whitethroat, tend to be sporadic and concentrated in rather few unpredictable rain events [39]. Hence, February rainfall in the wintering grounds would usually consist of one rain event. Additionally, the rain amounts mentioned in our study are averaged over a rather large region. Thus, given the sporadic characteristic of rainfall in this region, a higher mean precipitation value indicates a larger number of spots that have received any rain. Therefore, on years with higher mean precipitation amounts we would expect more birds to get the cue for taking off earlier, which will lead to an overall earlier median arrival time to Eilat.

Precipitation as a cue for phenological processes has been suggested by several Australian scientists who studied the onset of breeding and short distance migration of wetland birds [40–43]. In a study on breeding season onset of 49 Australian water bird species, Halse & Jaensch [40] found rainfall to be the most important proximate cue stimulating gonadal recrudescence. Saunders *et al*. [42] revealed strong association between egg-laying of the Carnaby's cockatoo, *Calyptorhynchus latirostris*, and autumn rainfall, and concluded that this tight synchrony indicates a strong reliance on early autumn rains as a cue for breeding. Chambers *et al*. [43] analysed 145 datasets of 52 Australian bird species. They reported significant shifts in timing of migration. Strength of phenological trends was strongly related to local climate variables, with precipitation, particularly number of wet days, being a major driver of movement in Australian birds. Chambers *et al*. interpreted this correlation as a cuing mechanism.

**Figure 3.**
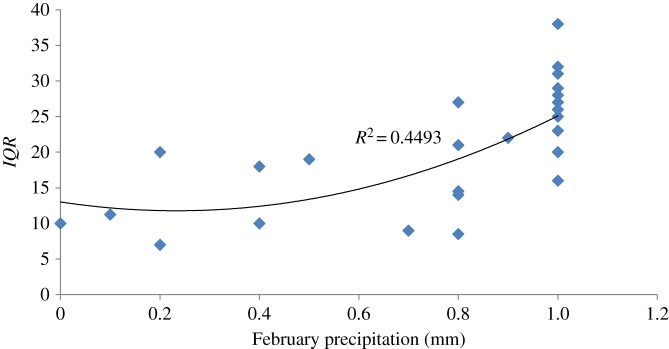
Interquartile range (*IQR*) of arrival day (*Calenday*) of the lesser whitethroat, *Sylvia curruca,* to Eilat as a function of February median precipitation in Africa, 1984–2013.

February median precipitation is by large the major variable involved in the model for the temporal spread (*IQR*) of spring migration. This variable exhibits a quadratic behaviour with opposite signs of the coefficient estimates of the first- and second-order parameters (figure 3). However, a careful examination of the function's mathematical behaviour indicates that it is mostly for the higher values of median February precipitation that the temporal spread of migration is larger. This outcome makes sense in light of the *Median Calenday* model; as stated above, the *Median Calenday* model predicts an earlier springtime departure under higher February rainfall levels, which would allow a potentially longer time slot for individual departure. This may lead to a greater variability among individuals. The other factor involved in the *IQR* model is yearly (March–February) precipitation. The coefficient estimate of this variable is positive, indicating larger variability in departure time among individual birds when yearly precipitation levels are greater. The wintering grounds of the lesser whitethroat are located in a mostly arid to semi-arid climate. Under these conditions, variability in precipitation patterns is usually large, and a rather small difference in yearly rainfall among sites may lead to a large variability in habitat structure and resource availability. Hence, it is likely that birds are not spread equally in the area but rather concentrate in those sites which are suitable in terms of food abundance. Moreover, precipitation patterns may vary between years and change site suitability. A rainy year should result in a larger number of suitable sites for wintering, which implies a larger overall variability in wintering conditions among wintering sites. That is, February precipitation variability among inhabited wintering sites should be larger on rainy years simply because of the larger number of sites occupied. This may be expressed in a larger variability in timing of spring migration among individuals.

Some other studies have examined patterns of spring migration of the lesser whitethroat. Horev *et al*. [44] studied correlations between temperature at wintering grounds and spring arrival in Eilat during 1985–2004 for three long-distance migrants, including the lesser whitethroat. They found no correlation of arrival time with temperature, which agrees with our own results. Sokolov & Kosarev [34] found high negative correlations between arrival time of the lesser whitethroat at Rybachy, Russia, and NAOI in February and March. Since NAOI is an index related to climate over parts of Europe, this observation strongly supports our proposition of February and March precipitation in Africa being a cue for the changing seasons. Tøttrup *et al*. [36] analysed spring arrival time during 1984–2004 to Eilat and Europe, of five migratory species, including the lesser whitethroat. They found delayed arrival in Eilat on years with high values of Normalized Difference Vegetation Index (NDVI, a remote sensing index of green reflection which indicates levels of primary production) at the wintering grounds prior to migration. This result contradicts the lack of any correlation in our data between annual or seasonal precipitation and spring arrival time of the lesser whitethroat. Moreover, if February precipitation would have affected NDVI, then, according to Tøttrup *et al.*'s results, the correlation between February precipitation and arrival time in Eilat should have been positive, and not negative as indicated by our results. The discrepancy between this study and our results may stem from two sources: first, the area considered as lesser whitethroat wintering grounds in Tøttrup *et al*.'s analysis was much smaller than the area we used in our study, probably as of accumulated data regarding the species winter distribution since. Second, the time period of our study was 10 years longer, which may produce a completely different outcome.

## 5. Conclusion

In this study, we examined the effect of climate conditions in the African wintering grounds of the lesser whitethroat on springtime arrival in Eilat, Israel, a major stopover site on the birds' flyway to European breeding grounds. Our results strongly suggest that February precipitation levels are the major factor dictating both timing of arrival and arrival spread along the season. As winter is dry season in that area, and since no other precipitation variables seem to correlate with arrival time, we propose that February precipitation serves as a cuing mechanism, fine-tuning the onset of spring migration. Seasonal air circulation and rainfall patterns in Africa support the feasibility of such a mechanism.

## Supplementary Material

Appendix I – Climate variables (46 variables: name, description, correlations with median and IQR of arrival day)

## Data accessibility

Bird data for this study were provided by the Israeli Birding and Research Centre in Eilat at: http://www.birds.org.il/en/bird-center-page.aspx?centerId=70. Climate data were downloaded from the University of East Anglia, UK, Climate Research Unit's website at: http://catalogue.ceda.ac.uk/uuid/3f8944800cc48e1cbc29a5ee12d8542d. The R Project free statistical software was used for analysis: https://www.r-project.org/.
