# Supplementary material for: February precipitation in the wintering grounds of the lesser whitethroat, Sylvia curruca: is it a cue for migration onset?
Source: R Soc Open Sci. 2017 Feb 15;4(2):160755. doi: 10.1098/rsos.160755 (PMC5367318; doi:10.1098/rsos.160755)
Supplement: Appendix I – Climate variables (46 variables: name, description, correlations with median and IQR of arrival day) [file rsos160755supp1.docx]

**Pearson’s correlation of climate variables with *Median Calenday***

| **Weather variables*** | **Correlation with *Median* *Calenday*** |
| --- | --- |
| Mean Temp Jan | 0.265393678 |
| Mean Temp Feb | -0.011910676 |
| Med Temp Feb | -0.035424617 |
| Mean Temp Mar | 0.184618147 |
| Med Temp Mar | 0.173326155 |
| Mean Max Temp Feb | 0.02067375 |
| Med Max Temp Feb | 0.003653247 |
| Mean Max Temp Mar | 0.237414961 |
| Med Max Temp Mar | 0.169128629 |
| Prop. sites w/Max Temp>35 Feb | -0.03518016 |
| Prop. sites w/Max Temp>35 Mar | 0.191436938 |
| Prop. sites w/Max Temp>38 Mar | 0.18980834 |
| Mean Min Temp Feb | -0.052096307 |
| Mean Min Temp Mar | 0.102712728 |
| Med Daily Temp Range Feb | 0.081184027 |
| Med Daily Temp Range Mar | 0.162808764 |
| Mean Cld Jan-Feb | -0.289686812 |
| Med Cld Jan-Feb | -0.314887008 |
| Mean Cld Nov-Feb | -0.314283316 |
| Med Cld Nov-Feb | -0.355928821 |
| Med Cld Feb | -0.343671425 |
| Med Cld Mar | -0.277447796 |
| Mean Hum Feb | -0.203399088 |
| Med Hum Feb | -0.102772665 |
| Mean Hum Mar | -0.098381177 |
| Med Hum Mar | 0.082191717 |
| Acc. Mean Pre Mar-Feb | 0.178761261 |
| Acc. Med Pre Mar-Feb | 0.049173689 |
| Acc. Prop Sites w >200mm Mar-Feb | 0.381695342 |
| Acc. Prop w >400mm Mar-Feb | 0.139125598 |
| Acc. Mean Pre Sep-Feb | 0.212062217 |
| Acc. Med Pre Sep-Feb | 0.280792628 |
| Acc. Mean Pre Dec-Feb | -0.178814129 |
| Acc. Med Pre Dec-Feb | -0.118176538 |
| Mean Pre Mar | -0.361103274 |
| Med Pre Mar | 0.036197368 |
| \| Mean Pre Jan \|  \| \| --- \| --- \| | 0.35908000 |
| Prop. Sites w/rain Jan | 0.309364924 |
| Prop. Sites w/rain Feb | -0.227625546 |
| Prop. Sites w/rain Mar | 0.053756287 |
| Mean Pre Feb | **-0.535159120** |
| Med Pre Feb | -0.038014507 |
| Mean Wet Nov-Feb | 0.374602773 |
| Mean Wet Jan | 0.462599771 |
| Mean Wet Feb | -0.359243372 |
| Mean Wet Mar | -0.334668125 |

Correlations which exceed an absolute value of 0.35 are marked in red.

* **Abbreviations used:**

Acc. – Accumulated

Cld – Cloudiness

Hum –- Air humidity

Max - Maximum

Med - Median

Min – Minimum

Pre – Precipitation

Prop. Sites w/ –proportion of sites with

Temp – Temperature

Wet – Number of wet days

For month names 3 first letters are used.

**Pearson’s correlations between IQR of *Calenday* and weather variables**

| **Weather variables** | | **Correlation**  **with *IQR*** | | |
| --- | --- | --- | --- | --- |
| Mean Temp Jan | | | 0.003684 |  |
| Mean Temp Feb | | | 0.271409 |  |
| Med Temp Feb | | | 0.274131 |  |
| Mean Temp Mar | | | 0.3332 |  |
| Med Temp Mar | | | 0.248971 |  |
| Mean Max Temp Feb | | | 0.271271 |  |
| Med Max Temp Feb | | | 0.276563 |  |
| Mean Max Temp Mar | | | 0.380036 |  |
| Med Max Temp Mar | | | 0.21893 |  |
| Prop. Sites w/Max Temp>35 Feb | | | 0.279843 |  |
| Prop. Sites w/Max Temp>35 Mar | | | 0.233036 |  |
| Prop. sites w/Max Temp>38 Mar | | | 0.280507 |  |
| Mean Min Temp Feb | | | 0.266795 |  |
| Mean Min Temp Mar | | | 0.247879 |  |
| Med Daily Temp Range Feb | | | 0.116873 |  |
| Med Daily Temp Range Mar | | | 0.314271 |  |
| Med Cld Feb | | | -0.06706 |  |
| Med Cld Mar | | | -0.24442 |  |
| Mean Cld Jan-Feb | | | -0.08978 |  |
| Med Cld Jan-Feb | | | -0.11400 |  |
| Mean Cld Nov-Feb | | | -0.21418 |  |
| Med Cld Nov-Feb | | | -0.23354 |  |
| Mean Hum Feb | | | 0.157895 |  |
| Med Hum Feb | | | 0.196243 |  |
| Mean Hum Mar | | | 0.228007 |  |
| Med Hum Mar | | | 0.206062 |  |
| Acc. Mean Pre Mar-Feb | | | 0.269271 |  |
| Acc. Med Pre Mar-Feb | | | 0.37154 |  |
| Acc. Prop w >200mm Mar-Feb | | | 0.426515 |  |
| Acc. Prop w >400mm Mar-Feb | | | 0.313042 |  |
| Acc. Mean Pre Sep-Feb | | | 0.257788 |  |
| Acc. Med Pre Sep-Feb | | | 0.246887 |  |
| Acc. Mean Pre Dec-Feb | | | 0.119612 |  |
| Acc. Med Pre Dec-Feb | | | 0.079989 |  |
| Mean Pre Mar | | | -0.24714 |  |
| Med Pre Mar | | | 0.194936 |  |
| Prop. Sites w/rain Jan | | | 0.34069 |  |
| Prop. Sites w/rain Feb | | | 0.440036 |  |
| Prop. Sites w/rain Mar | | | 0.171436 |  |
| Mean Pre Jan | | | 0.102546 |  |
| Mean Pre Feb | | | 0.137033 |  |
| Med Pre Feb | | | **0.629936** |  |
| Mean Wet Nov-Feb | | | 0.29138 |  |
| Mean Wet Jan | | | 0.209835 |  |
| Mean Wet Feb | | | 0.358652 |  |
| Mean Wet Mar | | | -0.25868 |  |
|  |  | | |  |
